# Clinical research progress of renal denervation for heart failure treatment: current evidence, controversies, and future directions

**DOI:** 10.3389/fcvm.2026.1790873

**Published:** 2026-03-06

**Authors:** Dan Zhang, Dong Wang, Xiaosu Wang, Jingdan Yu, Bo Liu

**Affiliations:** 1 Department of Clinical Laboratory, Wuhan Asia General Hospital Affiliated to Wuhan University of Science and Technology, Wuhan, China; 2 Department of Critical Care Medicine, Wuhan Asia General Hospital Affiliated to Wuhan University of Science and Technology, Wuhan, China

**Keywords:** catheter-based intervention, heart failure, HFpEF, HFrEF, neuromodulation, renal denervation, sympathetic nervous system

## Abstract

Heart failure (HF) management continues to evolve, yet morbidity and mortality remain high, particularly among patients with comorbid hypertension and heightened sympathetic activity. In this narrative review, we summarize the mechanistic rationale for renal sympathetic denervation (RDN) in HF, synthesize available clinical evidence across HF phenotypes, and highlight key controversies and future research priorities. We emphasize differential responses across geographic regions and consider how ongoing trials may refine patient selection, procedural strategies, and endpoints. As evidence grows, RDN may become an adjunct device-based therapy for selected HF patients, but definitive outcome trials are still needed before routine implementation.

## 1 Introduction

Heart failure (HF) represents the terminal stage of various cardiovascular diseases and has emerged as a major global public health challenge. According to recent epidemiological data, the worldwide prevalence of HF has reached approximately 64 million individuals, imposing substantial burdens on healthcare systems and societies (1). In China, the situation is particularly concerning, with the 2024 Chinese Heart Failure Guidelines reporting a prevalence rate of 1.3%, translating to approximately 13.7 million affected individuals (2). The aging population and improved survival rates following acute cardiovascular events have contributed to the continuously rising prevalence of this syndrome. Despite remarkable therapeutic advances over the past three decades, including the establishment of guideline-directed medical therapy (GDMT) with angiotensin-converting enzyme inhibitors, angiotensin receptor-neprilysin inhibitors, beta-blockers, mineralocorticoid receptor antagonists, and sodium-glucose cotransporter-2 inhibitors (3, 4), the prognosis of HF remains unsatisfactory. The five-year mortality rate continues to hover around 50%, comparable to many malignancies (5). Furthermore, current pharmacological approaches face several limitations: medication non-adherence affects 40%–60% of patients, drug intolerance restricts optimal dosing in a significant proportion, and substantial residual risk persists even among patients receiving optimal therapy (6). These unmet needs underscore the imperative for novel therapeutic strategies that can complement existing treatments. Comprehensive disease-modifying pharmacological therapy has been estimated to confer substantial lifetime benefit in HFrEF (7).

Sympathetic nervous system (SNS) overactivation constitutes a fundamental pathophysiological mechanism throughout the entire HF continuum (8, 9). In response to reduced cardiac output, compensatory neurohormonal activation initially maintains circulatory homeostasis but progressively becomes maladaptive, driving disease progression through direct cardiotoxic effects, promotion of cardiac remodeling, and perpetuation of the renin-angiotensin-aldosterone system (RAAS) cascade (10). The therapeutic value of targeting SNS hyperactivity has been validated by the survival benefits of beta-adrenergic receptor blockers in HF with reduced ejection fraction (HFrEF) (11, 12). However, pharmacological anti-sympathetic therapy has inherent limitations. Beta-blockers require gradual up-titration, may cause hemodynamic instability during initiation, and remain contraindicated or poorly tolerated in certain patient subgroups (13). More importantly, beta-blockers have failed to show mortality benefits in HF with preserved ejection fraction (HFpEF), despite evidence of SNS overactivation in this phenotype (14, 15). These limitations have stimulated interest in device-based neuromodulation approaches that could provide more targeted and sustained sympathetic attenuation without the constraints of daily medication adherence.

Renal denervation (RDN) represents a catheter-based intervention that ablates the sympathetic nerve fibers running along the renal arteries. Originally developed for treatment-resistant hypertension (16, 17), the concept of RDN has undergone substantial evolution. Following initial enthusiasm sparked by the SYMPLICITY HTN-1 and HTN-2 trials (18, 19), the field experienced a significant setback with the neutral results of the sham-controlled SYMPLICITY HTN-3 trial in 2014 (20). However, subsequent analyses revealed methodological limitations, including incomplete denervation and suboptimal patient selection, which prompted refinements in both procedural techniques and trial design (21). The resurgence of RDN has been marked by the positive results of the SPYRAL HTN and RADIANCE series, showing consistent blood pressure reductions in rigorously designed sham-controlled trials (22, 23). These findings have led to endorsement by the 2023 European Society of Hypertension Guidelines and the 2024 European Society of Cardiology Guidelines, positioning RDN as an option to be considered for patients with uncontrolled hypertension (24, 25). Recent regulatory approvals for catheter-based RDN in hypertension have further accelerated operator experience and program availability. The extension of RDN from hypertension to HF represents a logical therapeutic evolution, given the shared pathophysiological substrate of sympathetic overactivation. Koppe-Schmeisser and colleagues have conceptualized RDN as a potential “third pillar” of device therapy for HF, alongside cardiac resynchronization therapy and implantable cardioverter-defibrillators (26). This perspective has catalyzed a growing body of clinical research investigating the efficacy and safety of RDN specifically in HF populations.

This review focuses on HFrEF and HFpEF, as current RDN clinical studies have primarily targeted these two phenotypes. HFmrEF (heart failure with mildly reduced ejection fraction, LVEF 41%–49%), formally defined as a distinct category since 2016 (27), lacks dedicated RDN studies, although some HFrEF trials may have included patients with borderline ejection fractions. The absence of specific evidence for HFmrEF represents an acknowledged knowledge gap that warrants future investigation. The objectives of this comprehensive review are fourfold: first, to synthesize the current clinical evidence for RDN in both HFrEF and HFpEF; second, to critically analyze the ongoing controversies and debates in this evolving field; third, to identify key knowledge gaps that require addressing through future research; and fourth, to propose evidence-based recommendations for clinical practice and research priorities. Through this analysis, we aim to provide clinicians and researchers with a balanced perspective on the potential role of RDN in the contemporary HF therapeutic armamentarium.

This article is a narrative review synthesizing mechanistic and clinical evidence from published studies and publicly registered trials, without performing a formal systematic review or *de novo* meta-analysis.

## 2 Pathophysiological basis of RDN in heart failure

### 2.1 Sympathetic overactivation and the cardiorenal axis

The sympathetic nervous system (SNS) plays a central role in the pathophysiology of heart failure (HF). In response to diminished cardiac output, arterial baroreceptor unloading (high-pressure arterial baroreflex input from the carotid sinus and aortic arch) triggers compensatory SNS activation to maintain circulatory homeostasis (28). While initially adaptive, sustained sympathetic hyperactivity becomes profoundly maladaptive, directly contributing to disease progression through multiple mechanisms including increased myocardial oxygen demand, promotion of arrhythmogenesis, and acceleration of adverse cardiac remodeling (8).

The kidney occupies a central position in the cardiorenal sympathetic axis, functioning as both an effector and amplifier of sympathetic signaling (29, 30). Efferent renal sympathetic nerves stimulate renin release from juxtaglomerular cells, promote tubular sodium reabsorption, and reduce renal blood flow, thereby activating the renin-angiotensin-aldosterone system (RAAS) and exacerbating fluid retention (31). Conversely, afferent renal sensory nerves transmit signals to the central nervous system that further augment systemic sympathetic outflow, establishing a deleterious positive feedback loop (32). This bidirectional communication between the kidneys and the central sympathetic centers provides the mechanistic rationale for targeting renal nerves as a therapeutic strategy in HF. For comprehensive reviews of these mechanisms, readers are referred to recent publications by Lauder et al. (33) and Koppe-Schmeisser et al. (26).

Sympathetic activation in HF is commonly inferred using systemic surrogates (e.g., plasma norepinephrine, heart rate variability), and can be assessed more directly by microneurography-derived muscle sympathetic nerve activity (MSNA) and, where available, regional norepinephrine spillover techniques. However, most HF RDN trials have not incorporated serial MSNA or spillover measurements. In related populations such as resistant hypertension, reductions in sympathetic nerve firing after RDN support a central sympathoinhibitory effect (34), but whether similar changes occur across HF phenotypes remains an important evidence gap for future sham-controlled studies.

### 2.2 Mechanisms of RDN in heart failure

Renal denervation (RDN) achieves therapeutic effects through simultaneous ablation of both efferent and afferent renal nerve fibers (35). This dual denervation interrupts the pathological cardiorenal sympathetic circuit at a critical node, producing multifaceted benefits that extend beyond simple blood pressure reduction (33, 35, 36).

**Figure 1:**
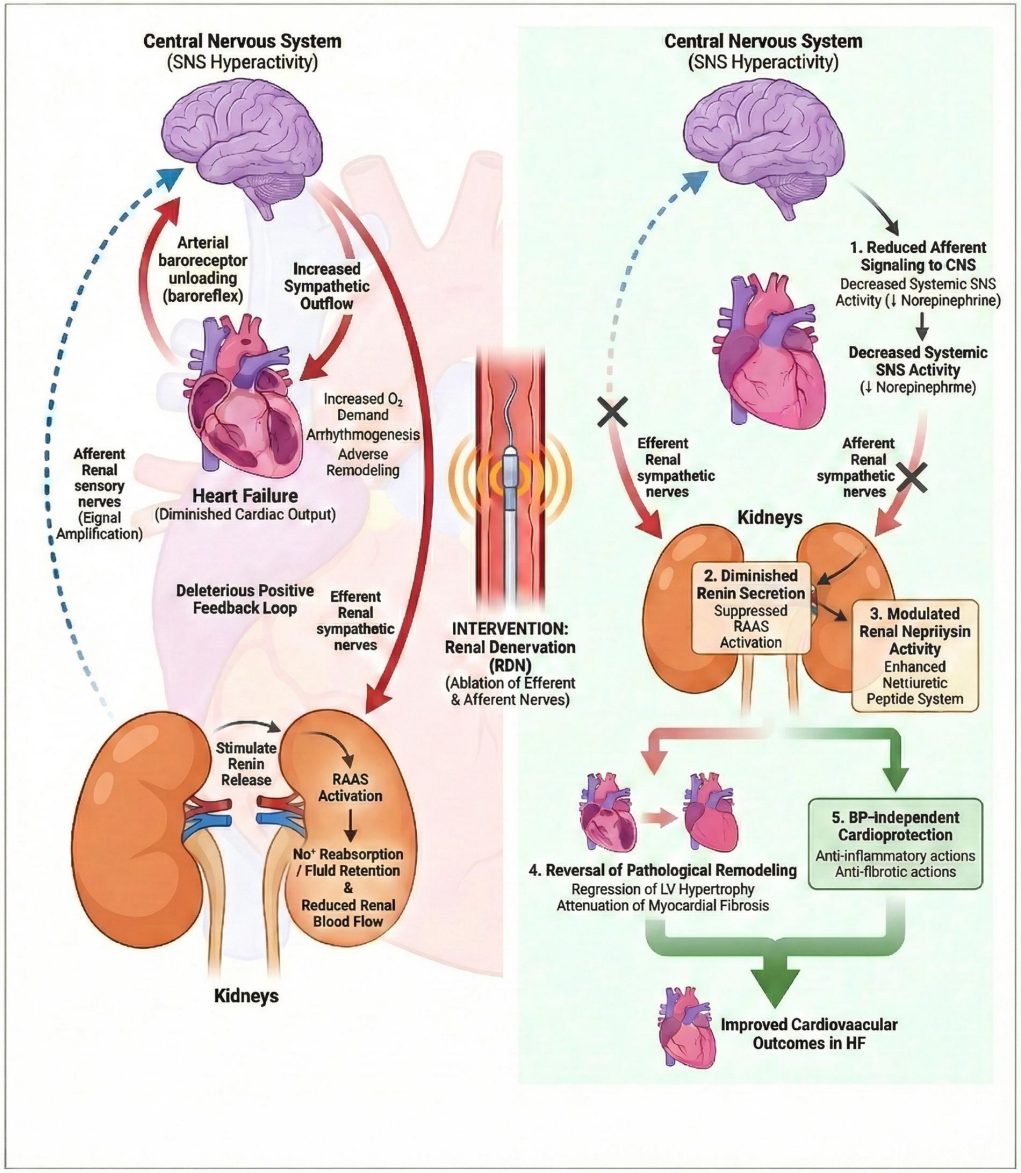
Targeting the vicious cardiorenal sympathetic cycle in heart failure via renal denervation (RDN). (Left panel) The vicious cardiorenal sympathetic cycle. In the setting of heart failure (HF), diminished cardiac output and arterial baroreceptor unloading (arterial baroreflex input from the carotid sinus and aortic arch) trigger a compensatory increase in central sympathetic outflow. Efferent renal sympathetic nerve activity (solid red arrows) is increased, stimulating renin release from juxtaglomerular cells, enhancing tubular sodium reabsorption, and reducing renal blood flow. This leads to activation of the renin-angiotensin-aldosterone system (RAAS) and fluid retention, which further exacerbates cardiac wall stress, oxygen demand, and adverse remodeling. Concurrently, afferent renal sensory nerve signaling (dashed blue arrows) transmits excitatory signals from the kidney to the central nervous system (CNS), further augmenting systemic sympathetic drive. This establishes a deleterious positive feedback loop that accelerates HF progression. (Right panel) Therapeutic mechanisms of renal denervation (RDN). The schematic illustrates disruption of the cardiorenal axis via catheter-based RDN. Simultaneous ablation of renal nerves (indicated by “X”) interrupts bidirectional communication between the kidney and the brain. (1) Ablation of afferent nerves reduces signaling to the CNS, thereby lowering systemic sympathetic tone and circulating norepinephrine levels. (2) Ablation of efferent nerves suppresses renin secretion and attenuates RAAS activation, mitigating fluid retention. (3) RDN may modulate renal neprilysin activity, potentially enhancing the bioavailability of cardioprotective natriuretic peptides. (4) Reduced sympathetic tone facilitates reversal of pathological cardiac remodeling (regression of LV hypertrophy and attenuation of myocardial fibrosis). (5) Emerging evidence suggests RDN exerts blood pressure-independent cardioprotective effects, including anti-inflammatory and anti-fibrotic actions, ultimately improving cardiovascular outcomes. CNS, central nervous system; LV, left ventricular; RAAS, renin-angiotensin-aldosterone system; SNS, sympathetic nervous system.

Five principal mechanisms underlie the potential cardiovascular benefits of RDN in HF. First, RDN reduces systemic sympathetic activity by eliminating afferent renal nerve signaling to the central nervous system, thereby decreasing circulating norepinephrine levels and attenuating direct catecholamine-mediated cardiotoxicity (30, 34). Second, ablation of efferent renal nerves diminishes renin secretion and subsequently suppresses RAAS activation, interrupting the neurohormonal cascade that drives HF progression (31). Third, emerging evidence suggests that RDN may modulate renal neprilysin activity. Polhemus and colleagues showed in preclinical models that RDN enhances the natriuretic peptide system through neprilysin-dependent mechanisms, potentially augmenting endogenous cardioprotective signaling (36). Fourth, sustained reduction in sympathetic tone facilitates reversal of pathological cardiac remodeling, including regression of left ventricular hypertrophy and attenuation of myocardial fibrosis (37, 38). Fifth, and perhaps most intriguing, accumulating data suggest that RDN may exert cardioprotective effects independent of blood pressure reduction. Lauder et al. have recently emphasized that the beneficial effects of RDN in HF may extend beyond hemodynamic improvements, encompassing direct anti-inflammatory and anti-fibrotic actions on the myocardium (33) (Figure 1).

### 2.3 Rationale for different HF phenotypes

The therapeutic rationale for RDN differs somewhat between HFrEF and HFpEF, though both phenotypes share the common substrate of sympathetic overactivation. In HFrEF, extensive evidence documents elevated plasma norepinephrine levels, increased muscle sympathetic nerve activity, and impaired heart rate variability, all of which correlate with adverse prognosis (9, 39). The established mortality benefit of beta-blockers in HFrEF validates the therapeutic importance of sympathetic modulation in this population (11, 40), and RDN offers a complementary device-based approach that may provide more sustained and complete sympathetic attenuation.

In HFpEF, sympathetic overactivation is similarly present but pharmacological anti-sympathetic therapy with beta-blockers has consistently failed to show clinical benefit (14, 15). This paradox may relate to the greater importance of arterial stiffness and ventricular-arterial coupling in HFpEF pathophysiology (41). RDN, by attenuating sympathetic tone and potentially improving vascular compliance, may address pathophysiological targets that are not adequately modulated by beta-blockade alone (42). Furthermore, RDN offers the advantage of a one-time intervention, circumventing the challenges of medication adherence that plague chronic pharmacotherapy. These considerations position RDN as a particularly attractive therapeutic option for HFpEF, a condition with substantial unmet therapeutic need (43, 44).

## 3 Clinical evidence for RDN in HFrEF

### 3.1 Early clinical investigations

The clinical investigation of renal denervation (RDN) in heart failure with reduced ejection fraction (HFrEF) began with the pioneering REACH-Pilot study (ClinicalTrials.gov registration number: NCT01584700) published in 2013 (45). This first-in-human investigation enrolled seven patients with chronic systolic HF (LVEF ≤ 40%) who underwent bilateral radiofrequency RDN. At six-month follow-up, the study showed significant improvements in six-minute walk distance (from 286 ± 57 meters to 353 ± 75 meters, *p* = 0.03) and patient-reported symptoms as assessed by the Minnesota Living with Heart Failure Questionnaire. Notably, there was no deterioration in renal function, addressing a critical safety concern given the importance of cardiorenal interactions in HF pathophysiology. While the study was limited by its small sample size and lack of a control group, it provided crucial proof-of-concept evidence that RDN could be safely performed in HFrEF patients and might confer symptomatic benefit. (NCT identifiers denote unique trial registration numbers on ClinicalTrials.gov.)

The subsequent REACH-Pilot extended study expanded upon these initial findings by extending follow-up to 12 months and showing sustained improvements in exercise capacity and quality of life metrics (46). More importantly, early echocardiographic data suggested potential improvements in left ventricular ejection fraction (LVEF), raising the hypothesis that RDN might favorably influence cardiac remodeling. These pilot observations provided the scientific rationale for larger, controlled investigations.

### 3.2 Controlled clinical trials

The IMPROVE-HF-I study represented a methodological advancement in evaluating RDN for HFrEF (47). This single-center, open-label randomized controlled trial at Erasmus MC randomized 50 patients with HFrEF (LVEF ≤ 35%, NYHA class ≥ II) in a 1:1 ratio to RDN plus optimal medical therapy (OMT) or OMT alone, with 6-month follow-up. The primary efficacy endpoint was change in iodine-123 meta-iodobenzylguanidine (^123^I-MIBG) heart-to-mediastinum ratio (HMR), a measure of cardiac sympathetic nerve activity. The study found no significant difference in ^123^I-MIBG HMR change between groups (*p* = 0.95), indicating that RDN did not significantly alter cardiac sympathetic tone at 6 months. The primary safety endpoint (composite of cardiovascular death, heart failure rehospitalization, and acute kidney injury) occurred in 8.3% vs. 8.0% of the RDN and OMT groups, respectively (*p* = 0.97). Secondary endpoints including LVEF, NT-proBNP, and quality of life measures showed no significant between-group differences. While IMPROVE-HF-I showed the safety of RDN in HFrEF, the neutral efficacy results highlighted the challenges of achieving meaningful sympathetic modulation with catheter-based approaches in this population. Other small randomized trials have reported improvements in remodeling and functional endpoints, while feasibility studies provide additional safety and follow-up signals (48–50).

### 3.3 Negative trials and methodological considerations

Not all planned clinical trials of RDN in HFrEF have been completed as designed. The RE-ADAPT-HF trial (NCT04947670) was designed as a prospective, multicenter, randomized, blinded, sham-controlled feasibility study to evaluate RDN in patients with chronic heart failure (LVEF < 45%, NYHA class II-III). The trial planned to enroll 144 patients and represented a methodological advancement by incorporating sham-control and blinded outcome assessment, addressing limitations of earlier open-label studies. However, the RE-ADAPT-HF trial was subsequently terminated due to slow enrollment, and no results have been published (51). The premature termination of this rigorously designed trial highlights the challenges of conducting sham-controlled device trials in heart failure populations and underscores the ongoing need for well-designed studies to definitively establish the efficacy of RDN in HFrEF (51, 52).

The divergent results across HFrEF trials have prompted systematic efforts to identify predictors of response to RDN. Analysis of pooled data from multiple studies suggests that patients with higher baseline blood pressure, elevated resting heart rate, and markers of sympathetic overactivation (such as reduced heart rate variability or elevated plasma norepinephrine levels) are more likely to derive benefit from RDN. Furthermore, procedural factors including complete circumferential ablation, treatment of branch vessels, and confirmation of adequate nerve injury appear to influence outcomes. These observations have informed the design of contemporary trials, which incorporate more stringent patient selection criteria and standardized procedural protocols. Emerging approaches, including machine-learning models, have been proposed to predict response to renal denervation (53).

### 3.4 Meta-analyses and pooled evidence

Meta-analyses provide an important synthesis of the available evidence for RDN in HFrEF. The comprehensive meta-analysis by Si et al. (Si F, Liu Q, Ma X, Yu J), published in Cardiorenal Medicine in 2025, identified 6 randomized controlled trials (RCTs) and 9 single-arm studies, totaling 352 participants with HFrEF (54). In the RCT analysis, RDN was associated with significant improvements in left ventricular end-diastolic diameter (LVEDD) [weighted mean difference (WMD) −3.55 mm, 95% CI −5.51 to −1.59, *p* < 0.01], left ventricular end-systolic diameter (LVESD) (WMD −4.13 mm, 95% CI −6.08 to −2.18, *p* < 0.01), and LVEF (WMD +6.30%, 95% CI 4.64%–7.96%, *p* < 0.01). Secondary analyses revealed significant benefits in six-minute walk test distance (WMD +51.25 m, 95% CI 8.30–94.20, *p* < 0.05) and BNP/NT-proBNP reduction (standardized mean difference −1.24, 95% CI −1.57 to −0.90, *p* < 0.01). Additionally, RDN significantly reduced heart rate (WMD −7.22 bpm, 95% CI −9.84 to −4.60, *p* < 0.01) without significant effects on systolic or diastolic blood pressure in HFrEF patients. The meta-analysis confirmed the safety of RDN in HFrEF patients, with no significant increase in adverse events including renal artery complications, deterioration of renal function, or symptomatic hypotension. It should be noted that the majority of included trials were open-label designs with relatively small sample sizes.

Heterogeneity in meta-analyses of RDN for HFrEF warrants systematic consideration, as it directly influences the strength of evidence and generalizability of findings. The Si et al. meta-analysis employed separate analytical approaches for RCTs and single-arm studies to address inherent methodological differences between study designs. Potential sources of heterogeneity include: (1) Study design differences—open-label trials may overestimate treatment effects compared to sham-controlled designs due to performance and detection bias; (2) Follow-up duration—ranging from 3 to 12 months across studies, with cardiac remodeling effects potentially requiring longer observation periods; (3) RDN technology and procedural variations—first-generation single-electrode catheters vs. second-generation multi-electrode systems (e.g., Symplicity Spyral) and ultrasound-based platforms (Paradise system) may achieve different degrees of denervation completeness; (4) Background guideline-directed medical therapy (GDMT)—variations in the proportion of patients receiving optimal beta-blocker, ACE inhibitor/ARB/ARNI, and mineralocorticoid receptor antagonist therapy across studies may influence the incremental benefit attributable to RDN; (5) Patient baseline characteristics—differences in age, baseline LVEF, NYHA functional class, etiology of heart failure, and presence of comorbidities (diabetes, chronic kidney disease, atrial fibrillation). Future meta-analyses should consider individual patient data approaches to better characterize treatment effect modifiers and identify responder phenotypes.

Exploratory subgroup analyses from the Si et al. meta-analysis revealed notable heterogeneity in treatment response based on geographic region. Studies conducted in Asian populations showed substantially greater LVEF improvement with RDN compared to non-Asian populations (WMD +7.08% vs. +0.86%, *p* for interaction <0.01) (54). However, this finding should be interpreted with caution given the *post-hoc* nature of the analysis and the potential for confounding by differences in trial methodology. Several hypotheses have been proposed to explain this geographic heterogeneity. First, genetic polymorphisms affecting sympathetic nervous system activity and responsiveness to denervation may differ between Asian and Western populations. Second, baseline characteristics including body mass index, renal artery anatomy, and prevalence of hypertensive heart disease may influence the therapeutic response to RDN. Third, differences in background medical therapy, including lower utilization of guideline-directed medical therapy in some Asian cohorts, may create a larger therapeutic window for RDN benefit. Fourth, methodological factors including the predominance of open-label designs in Asian studies may contribute to observed differences. This regional heterogeneity represents a critical knowledge gap that requires prospective validation in multinational trials with standardized protocols.

A recent review by Lauder and colleagues summarized mechanistic insights and the evolving clinical evidence for RDN in heart failure (33). Across available trials, exploratory analyses suggest that patients with higher baseline blood pressure, faster resting heart rate, and more pronounced sympathetic overactivation may derive greater functional and remodeling benefit, but these potential predictors require prospective validation (55, 56).

Beyond changes in functional status, available studies have reported structural cardiac adaptations after RDN, most commonly assessed by echocardiography (and in selected cohorts by cardiac magnetic resonance). Across pooled RCT evidence, improvements in LV dimensions (LVEDD/LVESD) and LVEF suggest reverse remodeling (54). However, the magnitude and durability of structural change may depend on baseline remodeling severity, denervation completeness, and concurrent GDMT optimization. Importantly, most HF RDN studies did not mandate structured exercise training or rehabilitation programs; therefore, observed improvements in exercise capacity should be interpreted as potentially attributable to RDN but may be influenced by unmeasured co-interventions, especially in open-label designs.

### 3.5 Comparison of RDN technologies in HFrEF

The evolution of RDN technology has influenced clinical outcomes in HFrEF trials. First-generation radiofrequency systems, exemplified by the Symplicity catheter, delivered point-by-point ablations primarily to the main renal artery trunk (55). Second-generation devices, including the Symplicity Spyral and EnligHTN systems, incorporated multi-electrode designs enabling more complete circumferential denervation and treatment of branch vessels. Ultrasound-based RDN, represented by the Paradise system, achieves denervation through targeted thermal injury without direct tissue contact, potentially offering more consistent lesion formation (56).

To date, no head-to-head randomized comparisons of different renal denervation (RDN) energy modalities (e.g., radiofrequency vs. ultrasound) have been conducted specifically in heart failure (HF) populations. Existing HF studies are generally small, open-label, and heterogeneous in device platforms and endpoints, which limits cross-study comparisons and highlights the need for standardized procedural protocols and sham-controlled designs (52).

### 3.6 Summary of key clinical trials

Importantly, robust event-driven, sham-controlled randomized trials showing that RDN improves hard outcomes (e.g., HF hospitalization or mortality) are still lacking. A feasibility sham-controlled program in HFrEF (RE-ADAPT-HF; NCT04947670) was terminated early because of slow recruitment, while an HFpEF program (UNLOAD-HFpEF; NCT05030987) is underway to test symptom and exercise haemodynamic endpoints. Together, these experiences underscore both the biological promise of sympathetic modulation and the practical challenges of delivering rigorous HF trials in this space (26, 51, 52).

Across published trials, RDN was generally delivered on top of usual care and contemporary background medical therapy; adjunct interventions such as supervised exercise training were not consistently mandated or reported (Table 1). Accordingly, future sham-controlled trials should prospectively standardize co-interventions and explicitly report medication titration and rehabilitation exposure to reduce confounding.

**Table 1:** Key published clinical studies of renal denervation (RDN) in heart failure with reduced ejection fraction (HFrEF).

Trial	Year	Design	Sample Size	Population	Primary Endpoint	Key Findings
REACH-Pilot	2013	Single-arm pilot study	7	NYHA II–III, LVEF <40%, on OMT	Safety/feasibility; functional status (6MWD), symptoms, renal function	Feasible and acceptable short-term safety; 6MWD improved (∼+27 m); no major renal safety signal reported
Symplicity HF Feasibility	2017	Prospective, multicentre single-arm feasibility study	39	NYHA II–III, LVEF <40%, on OMT	Safety; autonomic/sympathetic markers; symptoms and functional capacity	Acceptable procedural safety; no consistent improvement in LVEF or 6MWD; NT-proBNP decreased and functional class improved in some participants
IMPROVE-HF	2021	RCT, open-label (6-month follow-up)	50	HFrEF, NYHA II–III, LVEF ≤35%, on OMT	*Δ*^123^I-MIBG HMR	Neutral; No significant difference in ^123^I-MIBG HMR (*p* = 0.95)
Chen et al.	2017	Randomized, open-label pilot study	60	Chronic systolic HF, LVEF <40%	*Δ*LVEF at 6 months; 6MWD and biomarkers	Greater improvement in LVEF and 6MWD vs. control; NT-proBNP decreased; safety acceptable
Gao et al.	2019	Single-centre randomized controlled study (open-label)	60	Chronic systolic HF, NYHA II–III	Functional capacity and cardiac function (6MWD, LVEF) at 6 months	Improved 6MWD, LVEF and NT-proBNP vs. control; no major safety signals reported

RDN, renal denervation; HFrEF, heart failure with reduced ejection fraction; LVEF, left ventricular ejection fraction; 6MWD, 6-minute walk distance; NT-proBNP, N-terminal pro-B-type natriuretic peptide; MIBG, ^123^I-metaiodobenzylguanidine; HMR, heart-to-mediastinum ratio; NYHA, New York Heart Association; OMT, optimal medical therapy; eGFR, estimated glomerular filtration rate; SBP, systolic blood pressure. Trial registry IDs: NCT numbers are identifiers assigned by ClinicalTrials.gov; ChiCTR, Chinese Clinical Trial Registry.

Only published or publicly registered studies are included; absence of outcome data reflects ongoing or unpublished status rather than negative findings.

## 4 Clinical evidence for RDN in HFpEF

### 4.1 Pathophysiological rationale for RDN in HFpEF

Heart failure with preserved ejection fraction (HFpEF) represents one of the most challenging syndromes in contemporary cardiovascular medicine, characterized by substantial morbidity, impaired quality of life, and a paucity of evidence-based therapies (43, 44). Unlike HFrEF, where multiple pharmacological interventions have shown mortality benefits, no therapy has been shown to definitively reduce mortality in HFpEF, although SGLT2 inhibitors have emerged as the first class to significantly reduce heart failure hospitalizations in this population (57). The pathophysiology of HFpEF is complex and heterogeneous, involving diastolic dysfunction, ventricular-arterial stiffening, chronotropic incompetence, pulmonary vascular disease, and systemic inflammation (58). Importantly, sympathetic nervous system overactivation has been documented in HFpEF patients, with elevated plasma norepinephrine levels and increased muscle sympathetic nerve activity correlating with disease severity and adverse outcomes (59). However, these sympathetic phenotyping data are largely cross-sectional, and serial assessments before and after RDN in HF populations remain limited.

The rationale for RDN in HFpEF extends beyond isolated sympatholysis to encompass several pathophysiologically relevant mechanisms. First, arterial stiffness represents a central abnormality in HFpEF, contributing to increased afterload, impaired ventricular-arterial coupling, and exercise intolerance (41). RDN has been reported to improve vascular function and exercise capacity in selected HFpEF patients, potentially addressing this key pathophysiological derangement (42). Second, the frequent coexistence of resistant hypertension in HFpEF patients creates a therapeutic opportunity where RDN may simultaneously address blood pressure control and cardiac hemodynamics. Third, the failure of beta-blockers to improve outcomes in HFpEF suggests that pharmacological sympathetic blockade may not adequately target the relevant pathways, whereas device-based denervation may offer more comprehensive sympathetic modulation (14, 15). These considerations have prompted clinical investigation of RDN specifically in HFpEF populations.

Evidence gaps and mechanistic uncertainties: Although HFpEF cohorts demonstrate elevated systemic surrogates of sympathetic activation, direct quantification of organ-specific sympathetic nerve activity (cardiac, renal, or splanchnic) and organ-specific norepinephrine spillover remains limited. Therefore, much of the mechanistic rationale for RDN in HFpEF is inferential and should be interpreted in the context of marked HFpEF heterogeneity. In addition, renal nerves may partially reinnervate after ablation, and the relative contribution of interrupting renal afferent sensory signaling vs. reducing efferent renal sympathetic drive to clinical benefit is uncertain. Future HFpEF studies incorporating sympathetic phenotyping and longer-term follow-up are needed.

### 4.2 Clinical trial evidence

The only published randomized trial dedicated to HFpEF is the RDT-PEF study, an open-label, randomized, controlled pilot trial that enrolled 25 patients with symptomatic HFpEF. At 12 months, RDN did not show a consistent improvement in natriuretic peptides, echocardiographic indices of diastolic function, exercise capacity, or quality of life compared with control. Exploratory analyses suggested potential early signals in a subset of participants, but the study was underpowered and should be interpreted as hypothesis-generating rather than definitive evidence of efficacy (60, 61).

Beyond RDT-PEF, HFpEF evidence is largely limited to small mechanistic or observational cohorts, frequently enriched for coexisting resistant hypertension. These studies support biological plausibility (e.g., reduction in sympathetic tone and pulsatile ventricular afterload) but do not establish clinical efficacy in HFpEF (33, 42).

Beyond RDT-PEF, published clinical evidence for RDN in HFpEF remains limited, and no sham-controlled HFpEF trial has yet reported definitive clinical outcomes (Table 2). This underscores the need for rigorously blinded studies with standardized endpoints and systematic imaging follow-up of the renal arteries (26, 52). Earlier HFpEF-focused trial concepts and protocol publications include the DIASTOLE trial and the RESPECT-HF design report (62, 63).

**Table 2:** Published and ongoing studies of renal denervation (RDN) in heart failure with preserved ejection fraction (HFpEF).

Trial	Year	Design	Sample Size	Population	Primary Endpoint	Key Findings
RDT-PEF	2016	Open-label randomized controlled pilot trial (12-month follow-up)	25	Symptomatic HFpEF	Exploratory clinical/biomarker/echo outcomes (incl. natriuretic peptides, diastolic indices), exercise capacity and QoL	Underpowered; no consistent differences at 12 months; exploratory early signals reported in subsets; acceptable safety profile
Kresoja et al.	2021	Mechanistic/physiologic cohort study	NR	HFpEF (often with concomitant hypertension)	Haemodynamic/afterload and autonomic measures	Supports biological plausibility (sympathetic modulation and afterload effects); clinical efficacy remains unproven
DIASTOLE	2013	Randomized trial design (protocol publication)	Planned	HF with normal/preserved EF	Diastolic function, cardiac structure/function, functional capacity and symptoms	Design published (2013); trial terminated early due to recruitment difficulties; no outcome data published
UNLOAD-HFpEF	Ongoing	Single-center randomized, double-blind, sham-controlled pilot trial	Planned	HFpEF	Exercise haemodynamics (incl. PCWP) and symptom burden	Results pending
RESPECT-HF	2015	Phase II randomized trial design (protocol publication)	Planned	HF with normal/preserved EF	Cardiac structure/function, biomarkers, exercise and clinical status	Design published (2015); trial status unknown; no peer-reviewed outcome data available

RDN, renal denervation; HFpEF, heart failure with preserved ejection fraction; PCWP, pulmonary capillary wedge pressure; BNP, B-type natriuretic peptide; VO2peak, peak oxygen consumption; KCCQ, Kansas City Cardiomyopathy Questionnaire; E/e′, ratio of early mitral inflow velocity to early diastolic mitral annular velocity; LVMI, left ventricular mass index; LA, left atrium; LAVI, left atrial volume index; BP, blood pressure. Trial registry IDs: NCT numbers are identifiers assigned by ClinicalTrials.gov; other registries (e.g., ChiCTR) are country-specific trial registries.

Only published or publicly registered studies are included; absence of outcome data reflects ongoing or unpublished status rather than negative findings.

Accordingly, ongoing sham-controlled trials are essential. UNLOAD-HFpEF (NCT05030987) is designed to test whether RDN improves exercise pulmonary capillary wedge pressure (PCWP) and symptom burden in HFpEF. If mechanistic and symptomatic benefits are confirmed, subsequent trials powered for clinical events will still be required before HFpEF-specific recommendations can be made (26, 52).

### 4.3 Comparison with HFrEF evidence

When comparing the evidence base for RDN in HFpEF vs. HFrEF, several important distinctions emerge. First, the magnitude of LVEF improvement observed in HFrEF trials cannot be replicated in HFpEF by definition, as ejection fraction is preserved at baseline. This necessitates reliance on alternative endpoints such as diastolic function parameters, exercise capacity, and quality of life, which may be less sensitive to intervention effects or require longer follow-up to show change. Second, the heterogeneity of HFpEF as a syndrome creates challenges in patient selection and endpoint adjudication that are less pronounced in HFrEF. Subphenotyping approaches, including classification based on obesity status, atrial fibrillation burden, and pulmonary vascular disease, may help identify HFpEF patients most likely to benefit from RDN (64, 65).

Given the limited size and heterogeneity of the available HFpEF dataset, pooled quantitative estimates should be interpreted cautiously. Contemporary reviews converge on the view that robust conclusions in HFpEF await adequately powered, sham-controlled trials with standardized endpoints and careful phenotyping (26, 66).

### 4.4 Ongoing trials and future directions

Looking forward, HFpEF trials may benefit from enrichment strategies and deep phenotyping to identify patients most likely to respond, such as those with demonstrable sympathetic overactivity, resistant hypertension, or marked exercise-induced elevations in filling pressures. Adaptive trial designs may further improve feasibility in this heterogeneous syndrome (65, 66).

Endpoints should prioritize what matters to patients and clinicians: patient-reported outcomes (e.g., Kansas City Cardiomyopathy Questionnaire, KCCQ), functional capacity (peak VO2 and 6MWD), and mechanistic readouts (including invasive exercise PCWP), with longer-term follow-up for HF hospitalization and mortality where feasible (24, 26, 66).

In summary, RDN remains a biologically plausible but unproven intervention for HFpEF. Current data are insufficient to support routine clinical use, and future progress will depend on well-designed sham-controlled studies with rigorous haemodynamic, symptomatic, and (ultimately) event-driven endpoints.

Several ongoing initiatives may help clarify the clinical role of RDN in HF populations. UNLOAD-HFpEF (NCT05030987) is expected to inform whether RDN can improve exercise haemodynamics and symptom burden in HFpEF, while terminated and ongoing feasibility programs in HFrEF illustrate the logistical and methodological barriers (including recruitment, blinding, and endpoint selection) that must be addressed to enable larger event-driven trials.

Crucially, these trials should incorporate patient-centered measures (quality of life, functional capacity), physiologic readouts (exercise haemodynamics and autonomic biomarkers), and standardized procedural quality metrics to reduce variability in ablation completeness. The selection of primary endpoints should be aligned with phenotype (HFrEF vs. HFpEF) and the hypothesized mechanism of benefit.

In summary, evidence for RDN in HFpEF remains preliminary. The only published randomized pilot trial (RDT-PEF) was underpowered and did not show a consistent benefit at 12 months, while mechanistic and observational studies (often in HFpEF with concomitant resistant hypertension) support biological plausibility but cannot establish clinical efficacy. Ongoing sham-controlled studies, together with deeper phenotyping and standardized symptom and hemodynamic endpoints, will be essential before RDN can be considered for HFpEF outside clinical trials (26, 52, 60, 61, 66).

## 5 Safety and procedural considerations

### 5.1 Procedural safety profile

The safety of renal denervation (RDN) in heart failure (HF) populations has been a primary focus of clinical investigation, given the heightened vulnerability of these patients to procedural complications and hemodynamic perturbations. Reassuringly, data from both randomized controlled trials and real-world registries have consistently showed a favorable safety profile for RDN in HF patients, with complication rates comparable to those observed in hypertension trials (67). The Global SYMPLICITY Registry, which included a substantial subset of patients with concomitant HF, reported an overall major adverse event rate of 0.9% at 30 days, encompassing renal artery dissection, access site complications, and new-onset renal artery stenosis (68). Importantly, no significant differences in safety outcomes were observed between HF and non-HF subgroups, suggesting that the presence of HF does not substantially increase procedural risk.

Renal artery complications represent the most procedure-specific safety concern with RDN. Systematic analysis of pooled trial data reveals an incidence of renal artery stenosis or dissection of approximately 0.5% in contemporary studies utilizing second-generation devices (69). The risk of clinically significant renal artery stenosis requiring intervention is substantially lower, reported at 0.1%–0.2% in large registries. Procedural refinements, including the use of lower power settings, shorter ablation durations, and improved catheter designs, have contributed to the excellent vascular safety observed with current RDN technologies. Long-term imaging follow-up from the SPYRAL HTN and RADIANCE programs has confirmed the absence of late renal artery stenosis or aneurysm formation, providing reassurance regarding the durability of vascular safety (70).

### 5.2 Renal function considerations

The impact of RDN on renal function is of paramount importance in HF populations, where cardiorenal syndrome is prevalent and preservation of kidney function is a critical therapeutic goal. Most HF RDN trials have applied a baseline eGFR threshold of ≥30 mL/min/1.73 m² and excluded patients with advanced chronic kidney disease, reflecting both safety considerations and limits of available evidence. Periprocedural renal-protection strategies (e.g., minimizing contrast exposure, maintaining adequate perfusion pressure, and careful volume management) are particularly important in HF. Contrary to initial theoretical concerns that ablation of renal sympathetic nerves might compromise renal hemodynamics, clinical trial data have consistently shown stable or improved renal function following RDN in HF patients (71). Meta-analysis of HFrEF trials reveals no significant change in estimated glomerular filtration rate (eGFR) at 6–12 months post-procedure (weighted mean difference: +0.8 mL/min/1.73 m², 95% CI −1.2 to +2.8 mL/min/1.73 m²) (54). Several studies have reported modest improvements in renal function, potentially attributable to enhanced renal perfusion secondary to improved cardiac output and reduced neurohormonal activation.

The preservation of renal function following RDN in HF has important mechanistic implications. The renal sympathetic nerves, while contributing to pathological sodium retention and RAAS activation in HF, are not essential for maintenance of baseline renal hemodynamics. Compensatory mechanisms, including autoregulatory adjustments in renal vascular resistance and modulation of tubuloglomerular feedback, appear to maintain adequate renal blood flow following denervation. Furthermore, the reduction in systemic sympathetic activity and RAAS activation achieved by RDN may favorably influence glomerular hemodynamics and reduce proteinuria, as observed in hypertensive populations (72). These findings have important implications for patient selection, suggesting that moderate chronic kidney disease (eGFR 30–60 mL/min/1.73 m²) should not be considered a contraindication to RDN in HF patients. In practice, follow-up should include serial creatinine/eGFR assessment and (where relevant) proteinuria evaluation, particularly in patients with baseline chronic kidney disease or higher contrast exposure.

### 5.3 Hemodynamic considerations in HF patients

Heart failure patients present unique hemodynamic considerations during RDN procedures. Concerns regarding intraprocedural hypotension, contrast-induced nephropathy, and volume shifts during the procedure have prompted development of HF-specific procedural protocols. Contemporary approaches emphasize minimization of contrast volume through use of intravascular ultrasound guidance, maintenance of euvolemia through careful preprocedural optimization, and avoidance of excessive sedation that might compromise hemodynamic stability (73). The incidence of clinically significant intraprocedural hypotension requiring intervention has been reported at 2%–4% in HF trials, comparable to rates observed in general RDN populations.

Post-procedural blood pressure management requires particular attention in HF patients receiving RDN. While blood pressure reduction is a desired outcome, excessive hypotension may precipitate symptoms of low cardiac output or compromise renal perfusion. Clinical trials have reported a mean systolic blood pressure reduction of 8–15 mmHg following RDN in HF populations, which is generally well-tolerated and may permit down-titration of antihypertensive medications (74). However, careful monitoring during the early post-procedural period is essential to identify patients at risk for symptomatic hypotension. A gradual, individualized approach to medication adjustment, with attention to standing blood pressure and symptoms of orthostatic intolerance, is recommended in clinical practice guidelines.

### 5.4 Patient selection and procedural planning

Appropriate patient selection is critical for optimizing outcomes and minimizing procedural risk in RDN for HF. Based on current evidence and expert consensus, several patient-related factors should be considered when evaluating candidacy for RDN (75). Anatomical requirements include renal artery diameter greater than 3 mm, main renal artery length greater than 20 mm, and absence of significant renal artery stenosis (>50% luminal narrowing) or prior renal artery intervention. Patients with fibromuscular dysplasia, renal artery aneurysm, or severe renal artery tortuosity may not be suitable candidates for catheter-based RDN. Pre-procedural imaging with computed tomography angiography or magnetic resonance angiography is recommended to assess renal artery anatomy and exclude contraindications.

Clinical factors influencing patient selection include hemodynamic stability, renal function, and markers of sympathetic activation. Patients with decompensated HF should undergo stabilization and optimization of volume status prior to RDN. A minimum eGFR threshold of 30 mL/min/1.73 m² has been employed in most clinical trials, although this criterion may be relaxed in carefully selected patients with close nephrological monitoring. Evidence of sympathetic overactivation, including elevated resting heart rate, reduced heart rate variability, or elevated plasma norepinephrine levels, may identify patients most likely to benefit from RDN, although prospective validation of these selection criteria is ongoing. The presence of concomitant hypertension, while not required, may enhance the benefit-risk profile by providing an additional therapeutic target.

Heart failure stage and clinical stability should also be considered when selecting patients for RDN. Most published HF RDN studies have enrolled stable, ambulatory, symptomatic chronic HF (typically NYHA class II–III) receiving background guideline-directed medical therapy, whereas evidence is very limited in pre-HF/early-stage disease or in advanced, decompensated HF. Until stage-stratified data are available, we suggest that RDN be considered primarily in clinically stable patients after optimization of congestion status and medical therapy, and avoided during acute decompensation.

### 5.5 Procedural techniques and technologies

Contemporary RDN procedures in HF patients follow standardized protocols adapted from hypertension applications (76). Vascular access is typically obtained via the femoral artery, although radial access has been successfully employed with dedicated catheter systems. Following renal artery engagement with a guide catheter, the denervation catheter is advanced into the renal artery under fluoroscopic guidance. Radiofrequency-based systems deliver controlled thermal energy through electrodes positioned against the arterial wall, creating discrete lesions targeting the adventitial sympathetic nerve fibers. Treatment typically involves four to eight ablations in each main renal artery, with additional treatment of accessory renal arteries and branch vessels when anatomically feasible. Ultrasound-based systems achieve denervation through circumferential thermal injury without direct tissue contact, potentially offering more consistent lesion formation.

Procedural completeness has emerged as a critical determinant of clinical outcomes following RDN. *Post-hoc* analyses from hypertension trials have shown correlations between the number of ablation lesions, circumferential coverage, and blood pressure reduction (77). In HF populations, similar relationships between procedural completeness and cardiac outcomes have been suggested, although prospective data are limited. Emerging technologies for real-time assessment of denervation completeness, including measurement of renal artery wall temperature, impedance monitoring, and stimulation-based nerve mapping, hold promise for optimizing procedural efficacy and may be particularly valuable in HF patients where maximizing therapeutic benefit is essential.

While the safety profile of RDN in HF has been reassuring, several controversies and unresolved debates continue to shape the field. These ongoing discussions are essential for understanding the current limitations of evidence and guiding future research priorities.

## 6 Current controversies and debates

### 6.1 Endpoint selection and clinical relevance

A fundamental controversy in the RDN for heart failure (HF) field concerns the appropriate selection of primary endpoints for clinical trials. The majority of completed studies have employed surrogate endpoints, most commonly change in left ventricular ejection fraction (LVEF) for HFrEF trials and echocardiographic diastolic function parameters or exercise capacity measures for HFpEF trials. While these surrogates offer practical advantages including smaller sample sizes and shorter follow-up durations, their relationship to clinical outcomes that matter to patients—mortality, hospitalization, and quality of life—remains incompletely established (78). Critics argue that improvements in LVEF or six-minute walk distance, while statistically significant, may not translate into meaningful clinical benefit, particularly given the modest effect sizes observed in many trials.

At present, no event-driven, sham-controlled trial has established that RDN improves hard outcomes (HF hospitalization or mortality) in either HFrEF or HFpEF. Existing studies mainly show procedural feasibility and acceptable short-term safety, with inconsistent and often underpowered signals on surrogate endpoints such as natriuretic peptides, echocardiography, or functional capacity (26, 52).

### 6.2 Blood pressure-dependent vs. independent effects

A central mechanistic controversy concerns the extent to which the cardiac benefits of RDN in HF are mediated by blood pressure reduction vs. blood pressure-independent sympatholytic effects. This distinction has important implications for patient selection and outcome interpretation. Some investigators argue that the improvements in LVEF and symptoms observed in HF trials are primarily attributable to afterload reduction, suggesting that RDN may offer limited incremental benefit over optimized antihypertensive therapy (79). Supporting this perspective, several trials have shown correlations between the magnitude of blood pressure reduction and LVEF improvement following RDN.

Conversely, accumulating evidence supports the existence of blood pressure-independent cardioprotective effects of RDN. Lauder and colleagues have emphasized that RDN may favorably influence cardiac remodeling through direct anti-inflammatory and anti-fibrotic mechanisms that are not fully explained by hemodynamic changes (33). Preclinical studies have shown that RDN reduces myocardial fibrosis, attenuates inflammatory cytokine expression, and improves calcium handling in cardiomyocytes independent of blood pressure effects (80). Furthermore, clinical trials have reported significant improvements in cardiac parameters among HF patients with normal or well-controlled baseline blood pressure, suggesting that the therapeutic mechanism extends beyond simple afterload reduction. Resolution of this controversy will require mechanistic studies incorporating biomarkers of myocardial fibrosis, inflammation, and sympathetic activity alongside hemodynamic assessments.

### 6.3 Durability of therapeutic effect

The durability of RDN effects in HF represents another area of active debate. Theoretical concerns have been raised regarding the potential for sympathetic nerve regeneration following catheter-based denervation, which could lead to attenuation of therapeutic benefit over time (81). Animal studies have shown evidence of partial nerve regeneration within 6–12 months following RDN, although the functional significance of this regeneration remains unclear. Long-term clinical data from hypertension trials suggest sustained blood pressure reduction through 36 months of follow-up, providing some reassurance regarding durability (82, 83). However, extrapolation of these findings to HF populations requires caution, as the hemodynamic and neurohormonal milieu of HF may influence regeneration kinetics. In addition to neurobiological durability, long-term follow-up should include surveillance for delayed renal artery complications (e.g., stenosis), although contemporary registries suggest this risk is low with current-generation technologies (83).

Limited long-term data are available from HF-specific trials, with most studies reporting outcomes only through 6–12 months of follow-up. Extended follow-up from the REACH-Pilot cohort showed sustained improvements in functional capacity and quality of life through 24 months, although the small sample size precludes definitive conclusions (46). The question of whether repeat RDN procedures might be beneficial in patients with initial response followed by clinical deterioration remains unanswered. Registry data suggest that repeat procedures can achieve additional blood pressure reduction in hypertensive patients with suboptimal initial response, but similar data in HF populations are lacking. Prospective studies with extended follow-up and serial assessment of sympathetic activity are needed to definitively characterize the durability of RDN effects in HF.

Ongoing mechanistic and symptom-focused sham-controlled trials (e.g., UNLOAD-HFpEF) will be important for determining whether RDN can deliver reproducible benefits on exercise haemodynamics and patient-reported outcomes. However, even positive mechanistic results would still need to be followed by adequately powered event-driven trials before RDN could be considered for guideline-level indications in HF (26, 52).

Over the next several years, progress will likely hinge on: (i) rigorous HF phenotyping and enrichment strategies; (ii) standardized procedural endpoints to confirm ablation completeness; (iii) careful selection of primary endpoints aligned with mechanism; and (iv) pragmatic trial designs that enable recruitment and durable blinding (84).

Skeptics have raised concerns that the geographic heterogeneity may reflect publication bias, with positive results from Asian centers more likely to be published than negative results. Additionally, differences in background medical therapy, including lower utilization of guideline-directed medical therapy in some Asian cohorts, may create a larger therapeutic window for RDN benefit. The absence of sham-controlled trials from Asian populations limits the ability to exclude placebo effects as a contributor to the observed treatment responses (85). Conversely, proponents argue that biological differences in sympathetic nervous system activity, body composition, and renal artery anatomy may genuinely influence responsiveness to RDN. Resolution of this controversy requires prospective, multinational trials with standardized protocols and sham-control arms conducted across diverse geographic populations.

### 6.5 Position in the therapeutic algorithm

Perhaps the most practically important controversy concerns the appropriate positioning of RDN within the HF therapeutic algorithm. Current evidence does not support RDN as a replacement for guideline-directed medical therapy (GDMT), and all clinical trials have evaluated RDN as an adjunct to optimized pharmacological treatment. However, debate exists regarding whether RDN should be considered only in patients who remain symptomatic despite maximal GDMT, or whether earlier intervention might provide incremental benefit (86). Proponents of earlier intervention argue that sustained sympathetic overactivation contributes to disease progression and that earlier denervation might prevent irreversible myocardial damage. Skeptics counter that the evidence base for RDN in HF remains insufficient to justify intervention in patients who are adequately managed with pharmacological therapy alone.

Medication considerations deserve special attention. In hypertension populations, RDN may reduce blood pressure and can enable down-titration of antihypertensive medications in some patients; however, in HF populations, available studies have generally maintained guideline-directed medical therapy and were not designed or powered to test medication-sparing effects. Therefore, current evidence is insufficient to conclude that RDN reduces HF medication burden. Instead, any hemodynamic benefit (e.g., modest blood pressure lowering or improved congestion control) could theoretically facilitate tolerance and up-titration of disease-modifying HF therapies in selected patients, but this hypothesis requires prospective evaluation with standardized medication protocols. As an example, SGLT2 inhibitors exert cardiovascular benefit via multiple mechanisms beyond glucose lowering, including natriuresis and hemodynamic/neurohormonal effects (87).

The relationship between RDN and other device therapies (CRT, ICD) remains unexplored. The conceptualization of RDN as a “third pillar” of device therapy for HF (26) requires validation through comparative effectiveness research before informing clinical practice.

## 7 Knowledge gaps and future research directions

### 7.1 Need for clinical event-driven trials

The most critical evidence gap in the RDN for heart failure (HF) field is the absence of adequately powered randomized controlled trials evaluating clinical event endpoints, specifically cardiovascular mortality and HF hospitalization. While existing trials have shown consistent improvements in surrogate markers including LVEF, functional capacity, and neurohormonal biomarkers, these findings cannot be confidently extrapolated to predict effects on hard clinical outcomes (88). The regulatory approval of pharmacological therapies for HF has historically required demonstration of mortality or hospitalization benefit, and device-based interventions should arguably be held to similar evidentiary standards before widespread adoption can be recommended.

From a clinical standpoint, RDN should not be used with the primary intent of treating HF outside of clinical trials. In selected patients with HF and concomitant true resistant hypertension, RDN may be considered strictly in accordance with contemporary hypertension guidance, within experienced centers and with shared decision-making. Importantly, BP lowering in advanced HF should be approached cautiously, and medication optimization remains foundational (24, 25).

### 7.2 Identification of responder populations

A major knowledge gap concerns the prospective identification of patients most likely to benefit from RDN. *Post-hoc* analyses have suggested that patients with higher baseline sympathetic activity, elevated blood pressure, and faster resting heart rate derive greater benefit from RDN, but these observations require prospective validation (55, 89). The development of validated predictive algorithms incorporating clinical, imaging, and biomarker variables could enable personalized patient selection and improve the benefit-risk profile of RDN in HF populations.

Novel biomarkers of sympathetic activity hold promise for refining patient selection. Heart rate variability parameters, particularly the low-frequency to high-frequency ratio and time-domain measures such as SDNN, provide non-invasive assessment of autonomic balance that may predict response to RDN (90). Plasma biomarkers including norepinephrine, copeptin, and mid-regional pro-adrenomedullin reflect different aspects of neurohormonal activation and may complement clinical parameters in identifying optimal candidates. Imaging-based assessment of cardiac sympathetic innervation using ¹²³I-metaiodobenzylguanidine (MIBG) scintigraphy has shown promise in predicting response to device therapies in HF and warrants investigation in the RDN context (89). Integration of these multimodal assessments into composite risk scores represents a priority for future research.

### 7.3 Mechanistic understanding

Deeper mechanistic understanding of how RDN influences cardiac function in HF is needed to optimize therapeutic application. While the general principle of sympathetic modulation is well-established, the relative contributions of afferent vs. efferent nerve ablation, the importance of central vs. peripheral sympathetic effects, and the mechanisms underlying potential blood pressure-independent cardioprotection remain incompletely characterized (91). Translational studies incorporating invasive hemodynamic assessment, tissue sampling, and advanced imaging are needed to elucidate these mechanisms.

The interaction between RDN and guideline-directed medical therapy represents another area requiring mechanistic investigation. Whether RDN provides additive or synergistic benefit when combined with beta-blockers, RAAS inhibitors, and SGLT2 inhibitors, or whether it permits dose reduction of these agents while maintaining therapeutic effect, has important implications for clinical application (92). Pharmacokinetic and pharmacodynamic studies examining the interplay between device-based and pharmacological neurohormonal modulation could inform combination treatment strategies and optimize therapeutic synergy.

### 7.4 Technology development and procedural optimization

Technological advances in RDN delivery systems represent an important avenue for improving outcomes. Current limitations include uncertainty regarding completeness of denervation, lack of real-time procedural feedback, and variability in lesion quality between operators and devices. Emerging technologies for intraprocedural assessment of nerve ablation, including high-resolution mapping of renal nerve activity, stimulation-response testing, and imaging-based confirmation of lesion formation, hold promise for standardizing procedural technique and optimizing denervation completeness (93).

The development of HF-specific RDN protocols represents an additional priority, as current approaches are largely extrapolated from hypertension applications. Considerations including branch vessel treatment, accessory renal artery denervation, and combined renal-splanchnic nerve modulation warrant investigation (94). Novel energy modalities may offer advantages in specific configurations.

### 7.5 Health economic considerations

The cost-effectiveness of RDN in HF populations has not been systematically evaluated. While the one-time procedural cost of RDN is substantial, the potential for sustained benefit without ongoing medication requirements may favorably influence lifetime cost-effectiveness calculations (95). Reduction in HF hospitalizations, which represent the largest component of HF-related healthcare expenditure, could provide significant cost offsets. Health economic modeling studies incorporating trial-based efficacy data, real-world healthcare utilization patterns, and long-term outcome projections are needed to inform payer decisions and resource allocation.

Comparative effectiveness research examining RDN against alternative device therapies (cardiac contractility modulation, baroreflex activation therapy) will be essential for positioning RDN within the therapeutic landscape (96). Dedicated HF device registries capturing real-world outcomes will complement randomized trial evidence.

### 7.6 Evidence gap in HFmrEF

A notable gap in the current evidence base is the complete absence of dedicated clinical trials evaluating RDN specifically in heart failure with mildly reduced ejection fraction (HFmrEF, LVEF 41%–49%). This phenotype, formally recognized as a distinct category in the 2016 ESC Guidelines (27) and reaffirmed in subsequent updates, represents 10%–25% of the heart failure population and exhibits unique pathophysiological characteristics that bridge the HFrEF-HFpEF spectrum. The exclusion of HFmrEF from RDN research represents a significant limitation in understanding the full therapeutic potential of sympathetic modulation across the heart failure continuum.

The absence of HFmrEF-specific RDN trials likely reflects the recent formal recognition of this phenotype, historical inconsistencies in LVEF classification, and the pathophysiological heterogeneity of HFmrEF (including recovered and deteriorating phenotypes).

The rationale for investigating RDN in HFmrEF is compelling. Evidence suggests that sympathetic nervous system overactivation is present in HFmrEF, with plasma norepinephrine levels and muscle sympathetic nerve activity intermediate between HFrEF and HFpEF. Furthermore, recent pharmacological trials have shown that therapies effective in HFrEF, including SGLT2 inhibitors and sacubitril/valsartan, also provide benefit in HFmrEF populations, supporting the concept of a therapeutic continuum. Whether device-based sympathetic modulation through RDN would similarly extend across phenotypes represents an important unanswered question.

Future research should address this gap through retrospective analyses of existing trial databases, prospective registry studies, and dedicated randomized controlled trials in HFmrEF populations. Until such evidence becomes available, RDN cannot be recommended for HFmrEF outside of clinical research settings.

## 8 Clinical practice recommendations

### 8.1 Current guideline status

At present, renal denervation (RDN) for heart failure (HF) has not been incorporated into major international HF management guidelines, including the 2023 European Society of Cardiology (ESC) focused update and the 2022 American Heart Association/American College of Cardiology/Heart Failure Society of America (AHA/ACC/HFSA) guidelines (3, 4). This absence reflects the current evidentiary landscape, where improvements in surrogate endpoints have not yet been complemented by demonstration of clinical event reduction in adequately powered trials. However, the 2023 ESC Guidelines for the management of arterial hypertension and the 2024 ESC Guidelines for the management of elevated blood pressure and hypertension have endorsed RDN as a treatment option for uncontrolled hypertension, establishing a regulatory and clinical framework that may facilitate extension to HF indications as evidence matures (24, 25).

Expert consensus documents have begun to outline standards for patient selection, operator training, and procedural conduct for catheter-based renal denervation (RDN). The 2023 ESC Council on Hypertension and European Association of Percutaneous Cardiovascular Interventions (EAPCI) clinical consensus statement provides practical guidance for the use of RDN in hypertension and acknowledges potential expansion to other conditions characterized by sympathetic overactivity, including heart failure, while emphasizing that heart failure applications remain investigational (97). Similarly, the Heart Failure Association of the ESC has identified RDN as a promising investigational intervention warranting continued clinical research.

### 8.2 Proposed patient selection criteria

Based on the available evidence and expert opinion, we propose the following patient selection criteria for consideration of RDN in HF within the context of clinical trials or carefully monitored clinical practice at experienced centers. For HFrEF, suitable candidates should have LVEF ≤40%, NYHA functional class II-III symptoms despite at least three months of optimized guideline-directed medical therapy, and evidence of sympathetic overactivation as indicated by resting heart rate greater than 70 beats per minute in sinus rhythm or elevated plasma norepinephrine levels (98). Concomitant hypertension (systolic blood pressure >130 mmHg despite antihypertensive therapy) strengthens the indication by providing an additional therapeutic target. Patients should have suitable renal artery anatomy confirmed by pre-procedural imaging, estimated glomerular filtration rate greater than 30 mL/min/1.73 m², and absence of decompensated HF or recent hospitalization within four weeks.

For HFpEF, candidate selection in research settings typically focuses on patients with persistent symptoms and concomitant (often resistant) hypertension, with objective evidence of elevated filling pressures at rest or with exercise (e.g., invasive hemodynamics or echocardiographic indices) (42, 99). HFpEF diagnosis should be supported by structured, evidence-based diagnostic approaches such as the H2FPEF score when appropriate (99). Given the heterogeneity of HFpEF, patients with predominant vascular phenotypes characterized by arterial stiffness and impaired ventricular-arterial coupling may be most likely to benefit from RDN. Exclusion criteria for both HF phenotypes include severe renal artery stenosis (>50%), prior renal artery intervention, anatomically unsuitable renal arteries, active infection, and life expectancy less than one year due to non-cardiac comorbidities.

### 8.3 Procedural recommendations

RDN procedures in HF patients should be performed at centers with established expertise in both renal artery intervention and HF management, ideally within the framework of a multidisciplinary heart team including interventional cardiologists, HF specialists, and cardiac imaging experts (97). Pre-procedural evaluation should include comprehensive cardiac assessment (echocardiography, assessment of volume status, optimization of medical therapy), renal artery imaging (computed tomography or magnetic resonance angiography), and baseline assessment of sympathetic activity when available. Patients should be euvolemic and hemodynamically stable prior to the procedure.

Procedural conduct should follow established RDN protocols with attention to HF-specific considerations. Minimization of contrast volume through use of intravascular ultrasound guidance is recommended, particularly in patients with borderline renal function. Complete circumferential denervation of both main renal arteries should be targeted, with treatment of accessory renal arteries and branch vessels when anatomically feasible. Post-procedural monitoring should include observation for hemodynamic instability, assessment of access site complications, and renal function evaluation prior to discharge. Close follow-up at 1–2 weeks is recommended to assess for delayed hypotension and guide medication adjustment.

### 8.4 Post-procedural management and follow-up

Post-procedural management requires careful attention to blood pressure trends and potential need for antihypertensive medication adjustment. A gradual approach to medication reduction is recommended, with priority given to agents with less favorable tolerability profiles while maintaining guideline-directed HF therapies (100). Patients should be counseled regarding expected timelines for benefit, as improvements in cardiac function and symptoms may evolve over weeks to months following denervation. Standardized follow-up including echocardiography, functional assessment (six-minute walk test or cardiopulmonary exercise testing), and quality of life questionnaires at 3, 6, and 12 months enables systematic evaluation of treatment response.

Renal artery imaging at 6–12 months post-procedure is recommended to exclude delayed renal artery stenosis, although the incidence of this complication with contemporary RDN technology is very low. Patients who show favorable response to RDN should continue to receive optimized guideline-directed medical therapy, as RDN is intended to complement rather than replace pharmacological treatment. For patients without apparent benefit at 6–12 months, reassessment of the diagnosis and consideration of alternative interventions is appropriate, as the absence of response may indicate suboptimal patient selection, incomplete denervation, or alternative pathophysiology not amenable to sympathetic modulation.

## 9 Conclusion

Renal denervation (RDN) is a catheter-based neuromodulation therapy that targets the cardiorenal sympathetic axis, and it has been explored as an adjunct to guideline-directed medical therapy (GDMT) across the heart failure (HF) spectrum. Current evidence supports a generally acceptable procedural safety profile in HF cohorts, but follow-up imaging is inconsistent and sample sizes remain small. In HF with reduced ejection fraction (HFrEF), pooled analyses suggest potential improvements in cardiac remodeling, functional capacity, and biomarkers; however, the evidence base is dominated by small, often open-label studies, and effect estimates are vulnerable to bias and placebo effects. In HF with preserved ejection fraction (HFpEF), evidence is even more limited: the published randomized pilot trial has been neutral, and the field is awaiting robust, sham-controlled studies. Accordingly, key evidence gaps include confirmation of clinically meaningful patient-centered benefit, identification of responder phenotypes, durable mechanistic proof of sympathetic modulation, and event-driven outcomes. Until these gaps are addressed, RDN should be considered investigational in HF and restricted to well-designed clinical trials with rigorous phenotyping, sham control, standardized procedural quality metrics, and endpoints aligned with HF phenotype and mechanism. At present, renal denervation for heart failure should be confined to clinical trials or highly selected patients with concomitant resistant hypertension managed in experienced centers, pending robust sham-controlled outcome data.
