# Supplementary material for: Clinical research progress of renal denervation for heart failure treatment: current evidence, controversies, and future directions
Source: Front Cardiovasc Med. 2026 Mar 6;13:1790873. doi: 10.3389/fcvm.2026.1790873 (PMC13002592; doi:10.3389/fcvm.2026.1790873)

Supplementary Material

Clinical research progress of renal denervation for heart failure treatment:
current evidence, controversies, and future directions

Dan Zhang, Dong Wang, Xiaosu Wang, Jingdan Yu, Bo Liu

Frontiers in Cardiovascular Medicine | DOI: 10.3389/fcvm.2026.1790873

Supplementary Figure S1. Forest plot of pooled randomized controlled trial (RCT) effect estimates of renal denervation (RDN) in HFrEF for key outcomes (values reproduced from the meta-analysis summarized in Section 3.4).

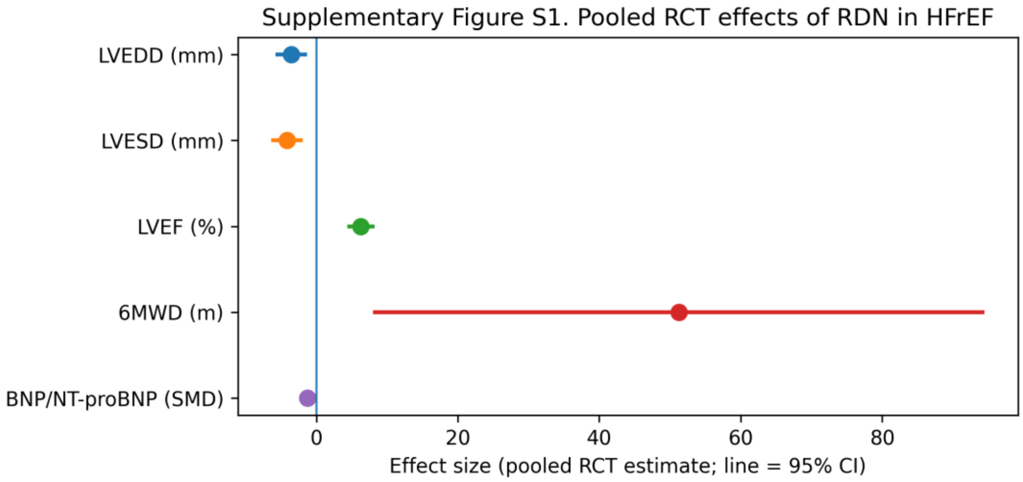

Supplement: Supplementary file 1 [file Image1.pdf]
